# Prevalence of dietary supplement use among male Brazilian recreational triathletes: a cross-sectional study

**DOI:** 10.1186/s13104-023-06665-9

**Published:** 2024-01-02

**Authors:** Vinnycius Nunes de Oliveira, Marilia Santos Andrade, Rafaella Sinisgalli, Rodrigo Luiz Vancini, Gustavo de Conti Teixeira Costa, Katja Weiss, Beat Knechtle, Claudio Andre Barbosa de Lira

**Affiliations:** 1 Faculdade de Educação Física e Dança, Universidade Federal de Goiás, Goiás, Goiânia Brazil; 2 Departamento de Fisiologia, Universidade Federal de São Paulo, São Paulo, Brazil; 3 Centro de Educação Física e Desportos, Universidade Federal do Espírito Santo, Vitória, Brazil; 4 Institute of Primary Care, University of Zurich, Zurich, Switzerland; 5 Medbase St. Gallen Am Vadianplatz, St. Gallen, Switzerland

**Keywords:** Triathlon, Cycling, Running, Swimming, Nutrition, Supplements

## Abstract

**Background and rationale:**

The literature shows that the prevalence of dietary supplements is high and guidance by a nutritionist or specialized professional is low in professional triathletes. It is reasonable to assume that in recreational triathletes, the prevalence of dietary supplements will also be high and that a significant portion of the sampled population will use supplements without any guidance from a qualified professional. The present study investigated dietary supplement use among Brazilian male recreational triathletes.

**Methods:**

A total of 724 Brazilian male recreational triathletes (age: 38.00 [10.00] years and body mass index: 24.16 [3.02] kg/m^2^) took part in this study. All participants answered an online questionnaire containing questions about their demographic characteristics and the nutritional aspects of their diet.

**Results:**

The results showed that ~ 90% (n = 653) of the interviewed participants reported using at least one dietary supplement. Surprisingly, ~ 25% did not receive supplement advice from a professional nutritionist.

**Conclusion:**

The prevalence of dietary supplements in male recreational triathletes was high, and a substantial part of the sample did not receive professional recommendations. This situation is worrisome because dietary supplements should be prescribed by a professional nutritionist.

**Practical implications:**

Our results suggest the need for an appropriate attitude and guidance by health professionals who deal with this population, especially nutritionists, to promote safe practices.

**Supplementary Information:**

The online version contains supplementary material available at 10.1186/s13104-023-06665-9.

## Introduction

Triathlon is an endurance sport that combines three modalities (swimming, cycling, and running) over various distances [1–4]. Numerous aspects have been studied, such as physiological, biomechanical, training, nutrition, morphology, and physical performance [4–11]. With regard to nutritional aspects, some triathletes have difficulties meeting the daily energy demand required by their training routine due to daily training commitments, residual training fatigue, reduced appetite after intense training sessions, lack of access to appropriate food and fluids, and family and work commitments, among others [12]. In these cases, it is necessary to supplement one’s nutrition and use dietary supplements [12]. Dietary supplements are products taken orally that contain an ingredient intended to supplement the standard diet [13]. The ingredients may include vitamins, minerals, herbs, amino acids, and carbohydrates [13].

Jovanov et al. investigated the prevalence of dietary supplement use and the source of information regarding supplementation in young athletes [14]. The authors found that the prevalence of dietary supplements used was 82.2%, and coaches were identified as the primary source of nutritional information. Wiens et al. also investigated the prevalence of dietary supplement use and the source of nutritional information among young Canadian athletes. The authors found that 98% of participants reported taking one or more dietary supplements and that the athletes received information on supplementation from family/friends (74%), coaches (44%), athletic trainers (40%), physicians (33%), and sports nutritionists (32%) [15].

Knez and Peake investigated the prevalence of dietary supplement use in ultra endurance triathletes and whether they used supplements under nutritional guidance. The authors found that the prevalence of supplements used was 62% and that in a sample of 37 triathletes, only one athlete used supplements under the guidance of a health professional (general practitioner) [16]. Therefore, the literature shows that the prevalence of dietary supplements is high and guidance by a nutritionist or specialized professional is low. Athletes who do not have the support of a professional multidisciplinary team find other sources of information [17]. It is important to highlight that this can leave the athlete vulnerable to disinformation and inappropriate recommendations, leading to health problems and performance impairment [13]. For example, a high sodium intake and inadequate water intake may contribute to the risk of kidney stone formation [18, 19]. Therefore, dietary supplements should be utilized as a complement to food intake and prescribed by a certified professional.

Therefore, it is reasonable to assume that in recreational triathletes, the prevalence of dietary supplements will also be high and that a significant portion of the sampled population will use supplements without any guidance from a qualified professional. To the best of our knowledge, little is known about the prevalence of dietary supplements used by recreational triathletes and whether they received adequate guidance from a nutritionist. Given that the number of triathlon practitioners is increasing, studies investigating the prevalence of dietary supplement use among recreational triathletes are warranted [6, 20]. The present study investigated dietary supplement use among Brazilian male recreational triathletes. We hypothesized that the prevalence of dietary supplements is high among recreational triathletes and that a significant portion of recreational triathletes use dietary supplements without guidance from a qualified professional.

## Methods

### Participants

The invitation to participate in the study was sent through email by the “Ironman Brazil company”. The survey was structured and applied using the Google Forms digital platform (See Supplementary Material) and was sent 30 days before the Ironman Brazil race was held (Florianopolis, Brazil, in May 2019). The athletes were instructed to answer the questions in the context of the week before completing the survey. The inclusion criteria were aged ≥ 18 years, male, literate, and familiar with online questionnaires. The exclusion criteria were incomplete or inconsistent replies to the questionnaire and being female. Initially, 1075 responses were received. Of the 1075 responses received, 351 subjects were excluded for not meeting the inclusion criteria of the study. Therefore, the final sample was composed of 724 respondents. The participant’s characteristics are described in Table 1. All experimental procedures were approved by the Human Research Ethics Committee of Federal University of Sao Paulo (approval number 3,318,080) and conformed to the principles outlined in the Declaration of Helsinki. All participants voluntarily gave their informed consent to participate in the study after having read the purpose of the study in the first section of the electronic survey.

**Table 1:** Characteristics of the participants

Variables	Men (n = 724)
	Median [IQR]^a^
Age (years)	38.00 [10.00]
Body mass (kg)	75.25 [12.00]
Height (m)	1.77 [9.00]
Body mass index (kg/m²)	24.16 [3.02]

^a^IQR: interquartile range

### Experimental procedures

To track the nutritional aspects, all participants answered a questionnaire. The questionnaire was composed of two sections. The first section included the following questions: name (open-ended question), sex (male or female), age (open-ended questions), body mass (open-ended questions), height (open-ended questions), and email address (open-ended questions). The second section included questions about nutritional aspects (use dietary supplements [Yes or No], dietary supplements utilized [open-ended question], nutritional guidance [Yes or No]), and professional area of nutritional guidance (nutritionist, sport physician, personal trainer, other specialists, nuthrologist, and others).

It is noteworthy that a validated questionnaire was not used, because there is no previously validated questionnaire focused on population studied (male Brazilian recreational triathletes). For this reason, we created a questionnaire. We tested questionnaire reproducibility through intraclass correlation coefficient (ICC). To this end, athletes answered the survey twice with one interval day. This analysis showed that ICC between the two surveys was classified as excellent for all questions. In this study, the second survey applied was used. The questionnaire did not have a finishing deadline and was written in Portuguese.

### Data analysis

A descriptive analysis was performed to summarize the data about the nutritional aspects. Descriptive data are presented as relative and/or absolute frequencies. The Shapiro-Wilk test was utilized to test the data normality of quantitative data. According to the Shapiro-Wilk test, the variables age, body mass, height, and body mass index did not present a normal distribution. Quantitative data were presented as medians and interquartile ranges. Intraclass correlation coefficient (ICC) was calculated to test the reproducibility of the questionnaire. ICC values less than 0.40 was classified as poor, between 0.40 and 0.59 as fair, between 0.60 and 0.74 as good, and between 0.75 and 1.00 was classified as excellent [21]. All data were analyzed through JASP (version 0.17.3.0, Netherlands).

## Results

**Fig. 1:**
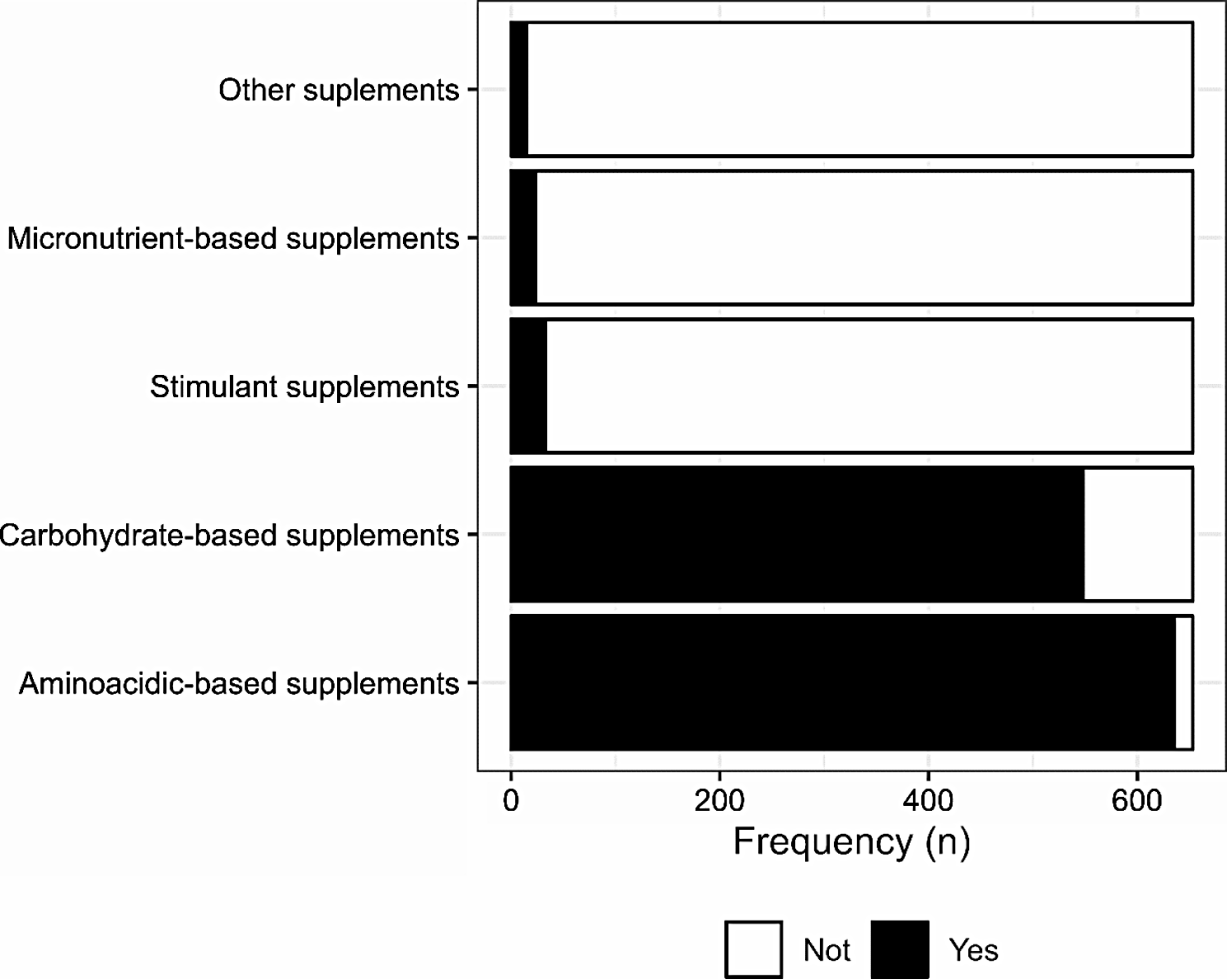
Use of supplements by categories

The prevalence of dietary supplements used was 90.2% (n = 653). Of the athletes evaluated that used dietary supplements, 97.2% (n = 635) reported taking aminoacidic-based supplements (for example, whey protein), 83.9% (n = 548) reported taking carbohydrate-based supplements, 3.5% (n = 23) reported using micronutrient-based supplements, 5.1% (n = 33) reported using stimulant supplements, and 2.3% (n = 15) reported using other supplements. These results are described in Fig. 1. It is noteworthy that the sum does not result in 100% because some athletes reported using two or more supplements.

**Fig. 2:**
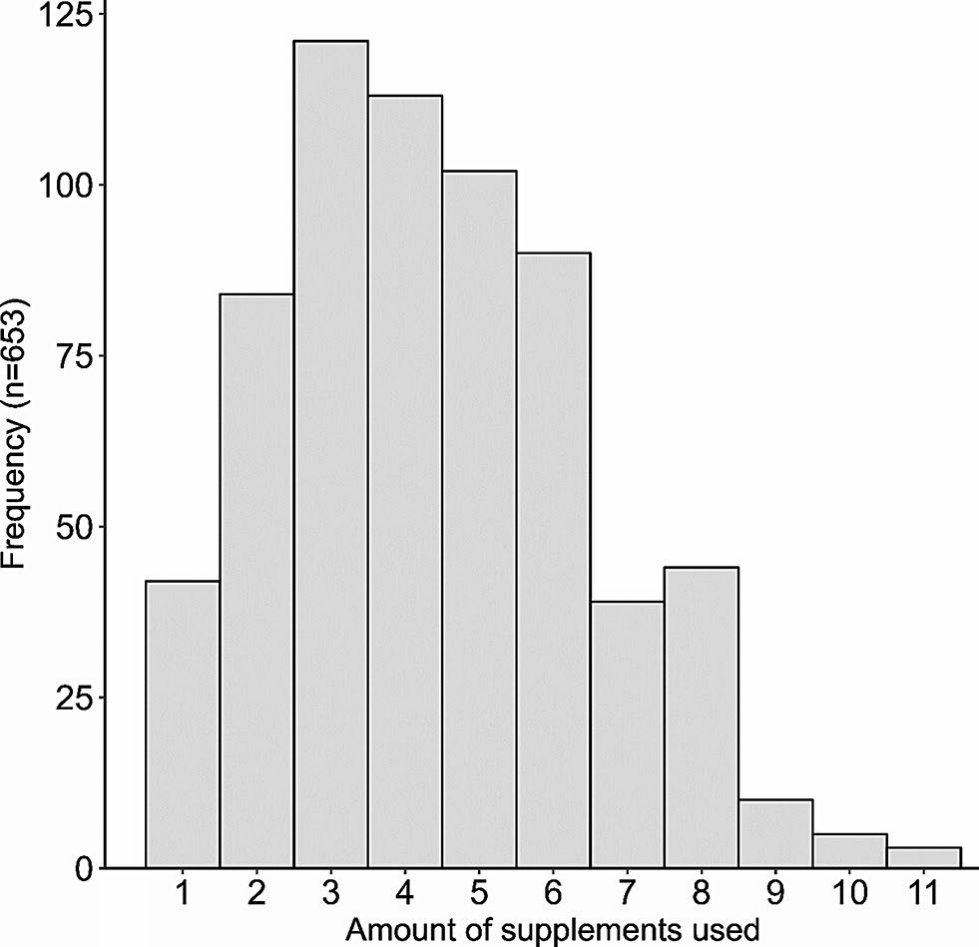
Amount of supplements used

Nevertheless, in relation to the dietary supplements, we found that 6.4% (n = 42) of the triathletes used only one dietary supplement, 12.9% (n = 84) used two dietary supplements, 18.5% (n = 121) used three dietary supplements, 17.3% (n = 113) used four dietary supplements, 15.6% (n = 102) used five dietary supplements, 13.8% (n = 90) used six dietary supplements, 6% (n = 39) used seven dietary supplements, 6.7% (n = 44) used eight dietary supplements, 1.5% (n = 10) used nine dietary supplements, 0.8% (n = 5) used ten dietary supplements, and 0.5% (n = 3) used eleven dietary supplements. These results are described in Fig. 2.

**Fig. 3:**
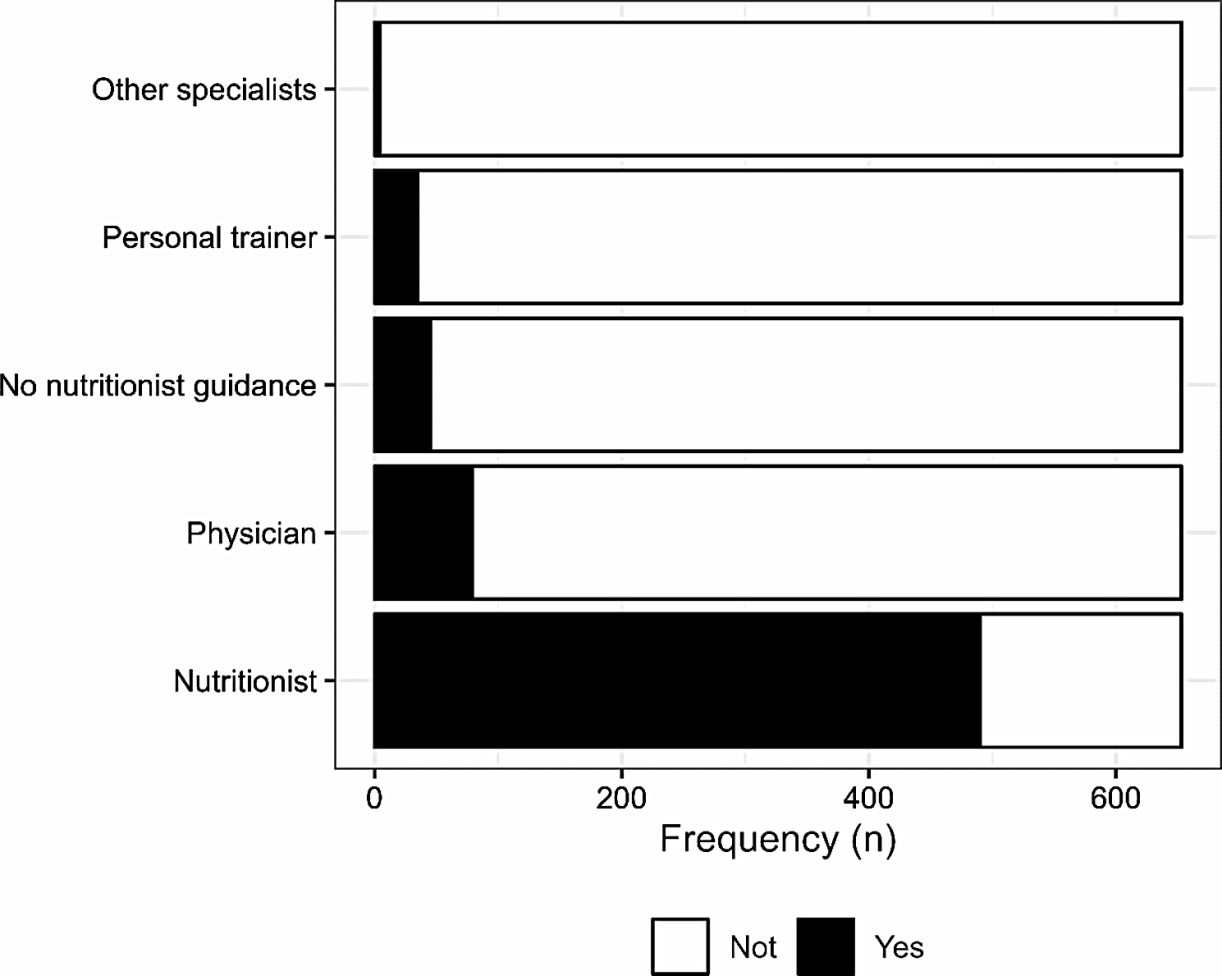
Nutritionist guidance

Of those who used supplements, 75% (n = 490) received guidance from a nutritionist, 12.1% (n = 79) received guidance from a physician, 6.9% (n = 45) used dietary supplements without nutritionist guidance, 5.4% (n = 35) received guidance by a personal trainer, and 0.6% (n = 4) received guidance by other specialists. Therefore, 25% (n = 163) did not receive any guidance from a professional nutritionist. These results are described in Fig. 3.

## Discussion

The aim of the present study was to characterize the nutritional aspects of recreational triathletes regarding their dietary supplement use. The findings were that the prevalence of supplement usage in this convenience sample was high, and part of the sample did not receive any nutritional guidance.

A possible explanation for the high supplement usage is the expansion of the dietary supplement industry, which impacts athletes who continually seek competitive advantage [13]. Furthermore, the literature shows that triathletes present a higher training volume. For example, elite triathletes commonly train more than 20 h/week [22]. High training volume is associated with high energy intake, and this condition is essential to health maintenance and physical performance in the triathlon [16]. In this context, dietary supplements can be utilized if food ingestion is not sufficient for some nutrients [23].

Froiland et al. found that the prevalence of supplement use in a sample of 207 college athletes (88 women and 115 men) was 89% (n = 184) [13]. Viana et al. examined the use of dietary supplements by Brazilian physical education professionals. The authors found that 49% of participants used dietary supplements and that the most-consumed supplements were rich in protein [23]. Vancini et al. investigated the prevalence and profile of dietary supplements and ergogenic aids among resistance training practitioners and found that 77% of the participants declared that they had already used dietary supplements and ergogenic aids. Whey protein (66%) and branched-chain amino acids (48%) were the most commonly used dietary supplements [24]. Graybeal et al. investigated the prevalence of dietary supplements use in endurance athletes (cyclists, runners, and triathletes). To this end, two-hundred cyclists, runners, and triathletes (females = 108) completed a questionnaire regarding the prevalence. Overall, 78.0% of athletes reported current supplement use. In addition, older athletes used more supplements than younger athletes. The majority of athletes (53.8%) used ≥ 3 supplements [25]. Another study found, in Spanish triathletes, that the consumption of supplements was high when compared to other sports disciplines, due to the high physiological requirements during training and competition [26]. Therefore, our results are in line with literature, because revealed that dietary supplement usage among Brazilian recreational triathletes also is high.

The current study also showed that 25% of the interviewed participants did not receive nutritional guidance by a professional nutritionist. This result is worrisome because it is desirable that dietary supplements are prescribed by nutritionists. Therefore, our results suggest the need for appropriate attitudes and guidance by health professionals who deal with this population, especially nutritionists, to promote safe practices. Froiland et al. concluded that athletes with access to a professional multidisciplinary team (i.e., a nutritionist) would consult them regarding nutrition and dietary supplementation [13].

Our study is not without limitations. First, it was a cross-sectional study; therefore, no causal relationship can be performed. Second, as we used a questionnaire, the results rely on the participants’ memory. Third, participants were asked to answer according to the previous week. Therefore, the conclusions are limited to this period. Fourth, the classification of the supplements used (aminoacidic-based, carbohydrate-based, micronutrient-based, stimulant supplements) did not follow previous classification made by organizations [27–29]. Nevertheless, we believe that these limitations do not prevent the conclusions of the study from being drawn.

In conclusion, the prevalence of supplements in recreational triathletes is high, especially protein-based supplements. However, a portion of the sample did not receive nutritional guidance by a certified professional. This is worrisome because of the high demands of triathlon from the organism in terms of physical and mental resources. Our results suggest the need for an appropriate attitude and guidance by health professionals who deal with this population, especially nutritionists, to promote safe practices.

### Supplementary Information

Below is the link to the electronic supplementary material.

**Additional File 1:** Data that support the findings of this study

## Declarations

### Disclaimer

Conclusions arise from the research are of the researchers.

## Abbreviations

ICC: Intraclass Coefficient Correlation
IQR: Interquartile Range
