# Supplementary material for: Prevalence of dietary supplement use among male Brazilian recreational triathletes: a cross-sectional study
Source: BMC Res Notes. 2024 Jan 2;17:8. doi: 10.1186/s13104-023-06665-9 (PMC10763403; doi:10.1186/s13104-023-06665-9)
Supplement: Supplementary file 1 — Additional File 1: Data that support the findings of this study [file 13104_2023_6665_MOESM1_ESM.pdf]

Name:

Age (years):

Body mass (kg):

Height (cm):

Sex:

( ) Female
( ) Male

Email: ______________________________

Do you use dietary supplements?

( )Yes
( )No

If yes, which one:

( ) Whey Protein
( ) Glutamine
( ) BCAA
( ) Creatine
( ) Beta Alanine
( ) Gel Carbohydrates
( ) Maltodextrin
( ) Palatinosis
( ) L- Carnitine
Other:____

Do you have any nutritional guidance?

( ) Yes
( ) No

Se sim, de qual área profissional? If yes, what is the vocational training of the professional who guides you?

( ) Nutritionist
( ) Physician
( ) Personal Trainer
Other:____
